# Platelets differentially modulate CD4^+^ Treg activation via GPIIa/IIIb-, fibrinogen-, and PAR4-dependent pathways

**DOI:** 10.1007/s12026-021-09258-5

**Published:** 2021-12-21

**Authors:** Matthias Bock, Christian B. Bergmann, Sonja Jung, Peter Biberthaler, Laura Heimann, Marc Hanschen

**Affiliations:** 1 Experimental Trauma Surgery, Klinikum Rechts Der Isar, School of Medicine, Technical University of Munich, Ismaninger Strasse 22, 81675 Munich, Germany; 2 Department of Cardiology, School of Medicine, German Heart Centre Munich, Technical University of Munich, Lazarettstr. 36, 80636 Munich, Germany; 3 Department of Trauma Surgery, Klinikum Rechts Der Isar, School of Medicine, Technical University of Munich, Ismaninger Strasse 22, 81675 Munich, Germany; 4 Division of Research, Department of Surgery, College of Medicine, University of Cincinnati, 231 Albert Sabin Way, Cincinnati, OH 45267 USA

**Keywords:** Thrombocytes, CD4^+^ regulatory T cells, Trauma, Glycoprotein IIb/IIIa, α_Iib_β_3_, Fibrinogen, Protease-activated receptor 4

## Abstract

CD4^+^FoxP3^+^ regulatory T cells (CD4^+^ Tregs) are known to dampen inflammation following severe trauma. Platelets were shown to augment their posttraumatic activation in burn injury, but the exact mechanisms remain unclear. We hypothesized that platelet activation mechanisms via GPIIb/IIIa, fibrinogen, and PAR4 have an immunological effect and modulate CD4^+^ Treg activation early after trauma. Therefore, C57Bl/6 N mice were injected with tirofiban (GPIIb/IIIa inhibition), ancrod (fibrinogen splitting enzyme), or tcY-NH_2_ (selective PAR4 antagonist peptide) before inducing a third-degree burn injury of 25% of the total body surface area. Changes in coagulation, and local and systemic CD4^+^ Treg activity were assessed via rotational thromboelastometry (ROTEM®) and phospho-flow cytometry 1 h post intervention. The inhibition of GPIIb/IIIa and fibrinogen locally led to a higher basic activity of CD4^+^ Tregs compared to non-inhibited animals. In contrast, PAR4 disruption on platelets locally led to an increased posttraumatic activation of CD4^+^ Tregs. Fibrinogen led to complete elimination of coagulation, whereas GPIIb/IIIa or PAR4 inhibition did not. GPIIb/IIIa receptor and fibrinogen inhibition increase CD4^+^ Tregs activity independently of trauma. Both are crucial for thrombus formation. We suggest platelets trapped in thrombi are unable to interact with CD4^+^ Tregs but augment their activity when circulating freely. In contrast, PAR4 seems to reduce CD4^+^ Treg activation following trauma. In summary, GPIIb/IIIa-, PAR4-, and fibrinogen-dependent pathways in platelets modulate CD4^+^ Treg baseline activity, independently from their hemostatic functionality. PAR4-dependent pathways modulate the posttraumatic interplay of platelets and CD4^+^ Tregs.

## Introduction

### Immune disbalance and CD4^+^ Treg activation in the early phase after trauma

As of today, trauma is still the leading cause of death for people under 45 years. Nine percent of deaths worldwide are attributed to trauma (Injury and Violence, The Facts, WHO, 2014: http://apps.who.int/iris/bitstream/10665/149798/1/9789241508018_eng.pdf). Furthermore, overall mortality in patients undergoing emergent surgery due to trauma is about 50% [1–3]. Tissue injury resulting from severe trauma leads to a massive release of damage-associated molecular patterns (DAMPs) in sterile conditions and pathogen-associated molecular patterns (PAMPs) in non-sterile conditions [4]. Both trigger a strong inflammatory reaction, associated with increased mortality due to the inability to balance the immune response [5] which can lead to an impaired, immune-related hemostasis [6] and multiple organ failure [5]. Recent studies showed a correlation between the hyper-acute changes of leukocyte counts (≤ 1 h) and the development of multiple organ dysfunction in trauma [7]. In past studies, we revealed that CD4^+^ Tregs instead of CD4 effector cells are activated within this timeframe after trauma [8]. Elevated levels of Tregs have also been identified in burn patients with detrimental outcome [9].

### Interaction of platelets and immune cells, especially CD4^+^ Tregs

Platelets interact with the innate and the adaptive immune system via pro-inflammatory cytokine release [10] and direct modulation of immune cell functions such as neutrophil NETosis [11] and reactive oxygen species (ROS) [12]. Platelets modify the proliferation of T cells as they augment CD4^+^ Treg response in general and promote Th1 and Th17 activity in a biphasic way in an experimental in vitro setting [13]. Although this study showed an effect in terms of proliferation and release of cytokines, it did not determine whether CD4^+^ Tregs showed increased activation through intracellular activation. We recently demonstrated that the absence of platelets leads to an impaired CD4^+^ Treg activity in an early phase after trauma in an in vivo model in mice [14].

### The GPIIb/IIIa receptor and fibrinogen in coagulation and inflammation

The integrin receptor GPIIb/IIIa is one of the most abundant receptors on the surface of platelets, with a large storage in the open canalicular system and the alpha-granules [15, 16]. They play a pivotal role in the hemostatic activation of platelets [17]. GPIIb/IIIa binds to VWF, fibronectin, vibronectin, CD40L, and most importantly to fibrinogen [18]. Fibrinogen allows platelets to link with each other via GPIIb/IIIb and form a stable clot formation in a flowing blood stream [15, 18]. The role of fibrinogen and the GPIIb/IIIa receptor in inflammation is not yet fully understood. In humans, GPIIb/IIIa is an essential amplifier of the FcyRIIa initiated aggregation of platelets in response to different gram-positive bacteria [19]. Mice do not express FcyRIIa [20] so it is unknown to which extent activation of GPIIb/IIIa leads to an altered immunologic function of platelets.

### The role of PAR4 in coagulation and inflammation

Platelet expressed PAR1/PAR4 receptors that are relevant for hemostasis in humans [18]. In mice, platelets express PAR3/PAR4 with PAR3 serving as a cofactor [18]. Several animal models using PAR4 KO mice exhibited that absence of PAR4 leads to prolonged bleeding after injury [21] but not spontaneously [22]. PAR4 on mice platelets is activated by thrombin in high concentrations and also through interaction with PAR3 in low concentrations [23, 24]. PAR4 is present on platelets, endothelial cells [25], smooth muscle cells [26], and leukocytes, including T cells [27] and Tregs [28]. There seems to be a considerable similarity in the expression of PAR4 in humans and mice [22]. Thrombin is known to be a very important, predominantly pro-inflammatory mediator which activates platelets via PAR3 and 4 in mice [29]. These findings suggest that the activation of platelets can modulate the immune response in inflammation.

### Summary introduction

In previous works, we proved that CD4^+^ Tregs and platelets interact in the early phase after trauma [14]. These findings underline that platelets are immunocompetent cells and modulate adaptive immune functions. It is, however, unknown what mechanisms are involved in the platelet–CD4^+^ Treg interaction. Recently, it was shown that TNFR2- and TLR4-dependant mechanisms are involved in the posttraumatic activation of CD4^+^ Tregs. We now hypothesize that impairment of specific platelet activation pathways, via GPIIb/IIIa, its ligand fibrinogen, and the PAR4 receptor, that are known to play a key role in the induction of hemostasis, also plays a role in the immunologic posttraumatic regulation and leads to modulated CD4^+^ Treg activation early after burn trauma.

## Material and methods

### Animals

We purchased C57BL/6 N mice from Chares River Laboratories, in Sulzfeld (Germany). Six- to eighth-week-old mice were acclimatized in the Center for Preclinical Studies (ZPF) at the University hospital Klinikum rechts der Isar, Munich (Germany). The animals were maintained in VAF facilities with strict regulations concerning feeding, temperature, humidity and water supply, and hour light/dark regimen. The experimental protocols were approved by the responsible Committee on the Ethics of Animal Experiments (Regierung von Oberbayern, Munich, Germany, Permit Number: 55.2–1-54–2532-164–12).

### Burn injury model

After shaving the dorsum, a third-degree burn injury was induced by exposing 25% of the total body surface area (TBSA) to 90 °C hot water for 9 s for burn and 20 °C hot water for sham treatment. Sufficient anesthesia was applied by injection of ketamine (200 mg/kg) and xylazine (10 mg/kg). Following injury, the mice were resuscitated with 1 ml 0.9% saline intraperitoneally. In previous experiments, a peak in zeta-chain-associated protein kinase 70 (ZAP-70) and PKC-θ, as well as its phosphorylated forms, was observed in CD4^+^ Tregs 30 min to 1 h post scald injury [8]. We therefore chose to euthanize and analyze these parameters mirroring CD4^+^ Treg activation after 1 h post injury. Until then, all animals remained under anesthesia.

### Experimental protocols

**Fig. 1:**
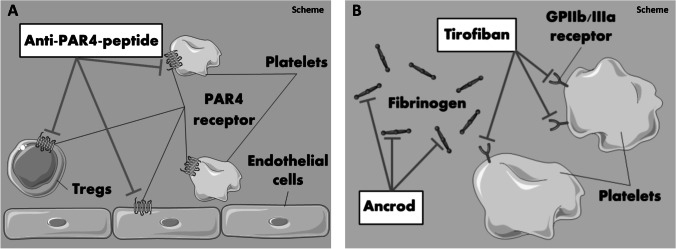
Schematic visualization of the experimental protocols. PAR4-dependent activation was blocked by usage of the selective PAR4 antagonist peptide tcY-NH2 (**a**). GP IIb/IIIa- and fibrinogen-dependent interaction of platelets and CD4^+^ Tregs was analyzed by usage of tirofiban and ancrod (**b**). The figure contains adapted graphics from Les Laboratoirs Servier—Medical Art under the terms of the Creative Commons Attribution License (CC BY) for non-commercial use. The use, distribution, or reproduction in other forums is permitted: https://creativecommons.org/licenses/by/3.0/legalcode, last accessed February 26, 2021

To measure modulating effects of GPIIb/IIIa, PAR4, and fibrinogen on the posttraumatic activation of CD4^+^ Tregs, we disrupted their functionality with three different drugs, which were injected 20 min prior to burn injury (Fig. 1). The injected drugs modulate fibrinogen- and thrombin-dependent pathways and displayed effective treatments in prior experimental protocols [30–32]. We therefore adhered to the established route and dosage of the named previous study protocols. In detail, glycoprotein IIb/IIIa inhibitor tirofiban (Aggrastat®, Correvio, London, UK) was applied intracardially (0.36 μg/g). The fibrinogen splitting enzyme, ancrod (Nordmark Arzneimittel GmbH & Co. KG, Uetersen, Germany), was injected intraperitoneally (i.p.) (8 IU). To assure continuous treatment, injection of ancrod was conducted twice (18 h and 20 min prior to burn injury). Selective PAR4 antagonist peptide, tcY-NH_2_ (Tocris Bioscience, Bristol, UK), was injected i.p. prior to burn trauma (0.6 mg/kg). The control groups were treated with saline intracardially and i.p. prior to trauma at the same time points.

To examine whether the effect is platelet dependent or mediated by the inhibition of PAR4 expressed on CD4^+^ Tregs, a spleen cell suspension of untreated mice was incubated with platelet-rich plasma (PRP) and stimulated with soluble anti-CD3ε Ab (1 µg/ml) (BioLegend, San Diego, CA) with and without the PAR4 antagonist peptide, tcY-NH_2_ (Tocris Bioscience), for 5 min.

### Reagents

Organs were harvested in C5 medium composed of the following solutions: RPMI 1640, HEPES buffer solution, 2-mercaptoethanol, L-glutamine 200 mM, 5% FBS, and penicillin/streptomycin (Sigma-Aldrich Chemical Company, St. Louis, MO) and MEM (GE Healthcare Bioscience Corp., Piscataway, NY). Cells were then washed with and stained in PBA buffer, composed of albumin bovine serum, sodium azide, and PBS (Sigma-Aldrich). Fc-Block (purified anti-CD16/32) (BioLegend) was added to prevent nonspecific binding. Fixation and permeabilization of cells were conducted using ice-cold methanol and diluted paraformaldehyde (PFA) (Carl Roth, Karlsruhe, Germany). Cells were labeled with anti-CD4 mAb (Miltenyi Biotec, Bergisch Gladbach, Germany) for surface staining and with anti-FoxP3 mAb (eBioscience, San Diego, CA) for detecting intracellular expression of FoxP3. Intracellular signal molecules were detected with the primary antibodies anti-PKC-θ (P632) and anti-p-PKC-θ (Thr 538), anti-ZAP-70 (99F2), and anti-p-ZAP-70 (Tyr 493) (Cell Signaling Technologies, Danvers, MA) followed by the labeled secondary antibody F(ab’)_2_ fragment of goat anti-rabbit IgG (Life technologies, Carlsbad, CA). Disruption of platelet functionality was conducted via tirofiban (Aggrastat, from Correvio, London, UK), which was injected intracardially prior to burn trauma. Another group was injected i.p. with ancrod (Nordmark Arzneimittel GmbH & Co. KG). The last group was injected i.p. with tcY-NH_2_ (Tocris Bioscience), a selective PAR4 antagonist peptide. Control groups were injected with saline.

### Cell processing (lymph nodes and spleen)

Euthanization of animals was conducted 1 h after burn injury and sham treatment by CO_2_ asphyxiation. Fourteen-gage needles were used to take blood samples intracardially 1 h following burn injury. Punctured blood was collected in a syringe with 3.1% trisodium citrate in blood citrate (ratio of 10:1). Draining lymph nodes (brachial, inguinal, and axillary) and spleen were harvested, minced, and strained with 70 µm pore sieves and washed in C5 buffer. Finally, all cells were stained and fixed with 0.15% PFA on 96-well plates.

### Phospho-flow cytometry

Cells were washed several times with PBA and permeabilized with ice-cold methanol (for 10 min). After application of a Fc-Block reagent (anti-CD16/32), anti-CD4 mAb and anti-FoxP3 mAb were added (incubation of 30 min at room temperature) to identify CD4^+^FoxP3^+^ regulatory T cells in phospho-flow cytometry. Primary antibodies (anti-PKC-θ (P632), anti-p-PKC-θ (Thr 538), anti-ZAP-70 (99F2), anti-p-ZAP-70 (Tyr 493)) were added and incubated for at least 30 min at room temperature. After washing, the labeled secondary antibody was added and incubated for 30 min at room temperature. The reactivity of the secondary antibody was tested in negative controls without application of the primary antibodies. Following several washing steps, the cells were fixed with 0.3% PFA, washed, and analyzed in PBS.

### Thromboelastometry

Following intracardial blood puncture, samples were analyzed via rotational thromboelastometry (ROTEM delta®) (Tem international GmbH, Munich, Germany). One hundred five microliters of mice blood was mixed with 7 µl of star-tem®/ex-tem®/fib-tem®. Impact of injected reagents on thrombin-, fibrinogen-, and PAR4-dependent pathways was observed by analyzing the following hemostatic parameters: clotting time (CT), α-angle, and maximum clot firmness (MCF).

### Statistics

We used the GraphPad Prism 9.0.1 software (GraphPad Software, Inc., LaJolla, CA) for statistical analysis. Groups were tested for normality and outliers. ANOVA on ranks, Kruskal–Wallis test, and Dunn’s multiple comparison were applied. Box plots represent median Q1 and Q3 values and mean values ± are shown. A *p* value of < 0.05 was considered significant.

## Results

### Basic CD4^+^ Treg activity within draining lymph nodes is elevated after impairment of GPIIb/IIIa and fibrinogen

**Fig. 2:**
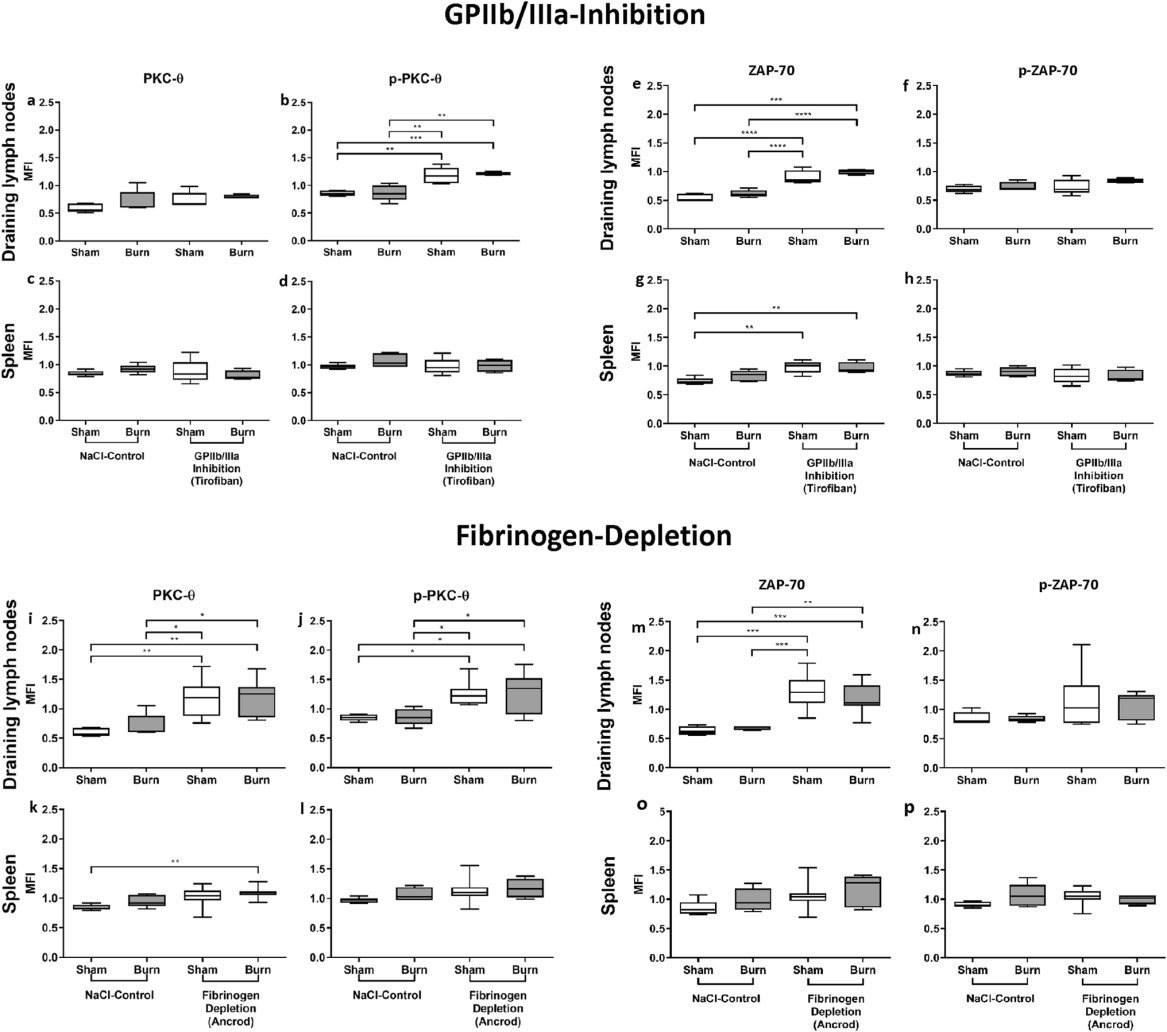
Basic CD4^+^ Treg activity is elevated after impairment of GPIIb/IIIa and fibrinogen. The inhibition of the platelet GPIIb/IIIa receptor and its ligand fibrinogen lead to a higher basic activity of CD4^+^ Tregs locally in the dorsum draining lymph nodes, without changing their posttraumatic activation. Systemically in the spleen, GPIIb/IIIa and fibrinogen disruption revealed a trend to increase basic activity of CD4^+^ Tregs. Data are expressed as box plots of the median fluorescent intensity (MFI) ± Q1 and Q3, and whiskers indicate max and min data points. *n* = 5–9. *p < 0.05, **p < 0.01, ***p < 0.001, ****p < 0.0001

In the first set of experiments, we addressed whether GPIIb/IIIa- and fibrinogen-dependent interactions between platelets (Fig. 1) have an effect on the posttraumatic activation of CD4^+^ Tregs. PKC-θ is an essential downstream signal molecule in the activation cascade of TCR signaling in T cell activation and its increase displays CD4^+^ Treg activity [14, 33]. We therefore analyzed it via phospho-flow cytometry to observe the activation of CD4^+^ Tregs 1 h following burn trauma (Fig. 2a–d). As a second marker for activation, we measured the expression of zeta-chain-associated protein kinase 70 (ZAP-70) and its phosphorylated form (Fig. 2e–h). ZAP-70 is also known to be part of the downstream signaling proteins of TCR [34]. Phosphorylated PKC-θ was increased in CD4^+^ Tregs in lymph nodes of mice treated with tirofiban and ancrod and in case of the latter also the non-phosphorylated form (Fig. 2b, i, j). Consistent with these findings, the basic expression of ZAP-70 was increased in the draining lymph nodes (Fig. 2e, m) but not their phosphorylated form (Fig. 2f, n). Collectively, the disruption of GPIIb/IIIa and fibrinogen led to significant higher basic activity of CD4^+^ Tregs in the draining lymph nodes. Interestingly, the inhibition of GPIIb/IIIa and fibrinogen did not modulate the posttraumatic expression and phosphorylation of PKC-θ and ZAP-70 in CD4^+^ Tregs in lymph nodes.

The expression and phosphorylation of PKC-θ did not differ significantly in CD4^+^ Tregs in the spleen of mice treated with tirofiban (Fig. 2c, d). Posttraumatic activation of splenic CD4^+^ Tregs in fibrinogen-inhibited mice was significantly increased compared to sham-untreated mice (Fig. 2k). Comparison of splenic posttraumatic CD4^+^ Treg activity versus sham in the fibrinogen-inhibited and in the control group showed just an increasing trend (Fig. 2k, l). However, ZAP-70 showed a significant increase in basic activity of CD4^+^ Tregs in the spleen after being treated with tirofiban (Fig. 2g) and an increasing trend in the fibrinogen-inhibited groups (Fig. 2o). We therefore conclude that the GPIIb/IIIa inhibition did not or to a minor extend change the basic activity systemically, as seen in the spleen compared to non-treated animals.

### PAR4 disruption leads to an increased posttraumatic activation of CD4^+^ Tregs within the draining lymph nodes

**Fig. 3:**
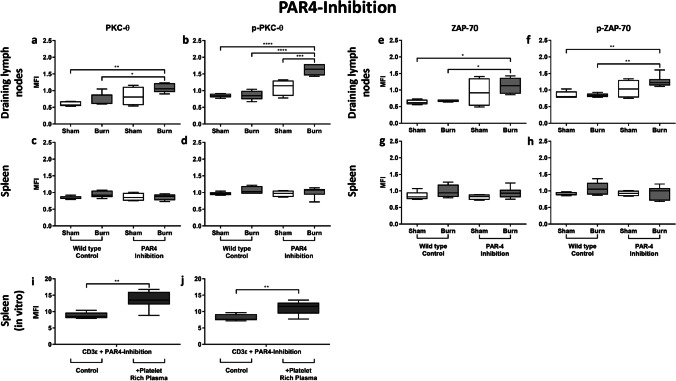
PAR4 disruption leads to a platelet-dependent increased posttraumatic activation of CD4^+^ Tregs in the draining lymph nodes but not systemically. The disruption of PAR4 led to increased posttraumatic activation of CD4^+^ Tregs in the draining lymph nodes, but not systemically in the spleen. CD4^+^ Treg activity in the dorsum draining lymph nodes (**a**, **b**, **e**, **f**) and the spleen (**c**, **d**, **g**, **h**) was measured after 1 h via phospho-flow cytometry by assessing the expression of phosphorylated and non-phosphorylated PKC-θ and ZAP-70. The effect was shown to be platelet dependent in vitro: a spleen cell suspension of untouched mice was incubated with platelet-rich plasma (PRP) and stimulated with CD3ε with or without the PAR4 antagonist peptide, tcY-NH_2_ (**i**, **j**). Data are expressed as box plots of the median fluorescent intensity (MFI) ± Q1 and Q3, and whiskers indicate max and min data points. *n* = 4–6 (**a**–**h**); *n* = 6 (**i**, **j**). *p < 0.05, **p < 0.01, ***p < 0.001, ****p < 0.0001

The PAR4 receptor is expressed on platelets, CD4^+^ Tregs, and the endothelium, facilitating cell communication in inflammatory settings (Fig. 1). PAR4 inhibition led to a significantly higher posttraumatic expression and phosphorylation of PKC-θ in CD4^+^ Tregs in the draining lymph nodes (Fig. 3a, b). Although the same trend was observed with ZAP-70, the changes did not reach the threshold of statistical significance (Fig. 3e, f). Expression and phosphorylation of PKC-θ did not differ significantly in CD4^+^ Tregs in the spleen following PAR4 inhibition (Fig. 3c, d), nor did ZAP-70 expression and its phosphorylated form (Fig. 3g, h). Therefore, neither basic activity nor posttraumatic activation of CD4^+^ Tregs was modulated by PAR4 inhibition in the spleen. To examine whether the effect is platelet dependent or mediated by the inhibition of PAR4 expressed on CD4^+^ Tregs, a spleen cell suspension of untreated mice was incubated with platelet-rich plasma (PRP) and stimulated with soluble anti-CD3ε Ab. We observed a significantly higher expression and phosphorylation of PKC-θ following PAR4 inhibition in the presence of platelets (Fig. 3i, j), indicating a platelet-dependent mechanism.

### Early posttraumatic hemostasis is not changed after GPIIb/IIIa inhibition via tirofiban

**Fig. 4:**
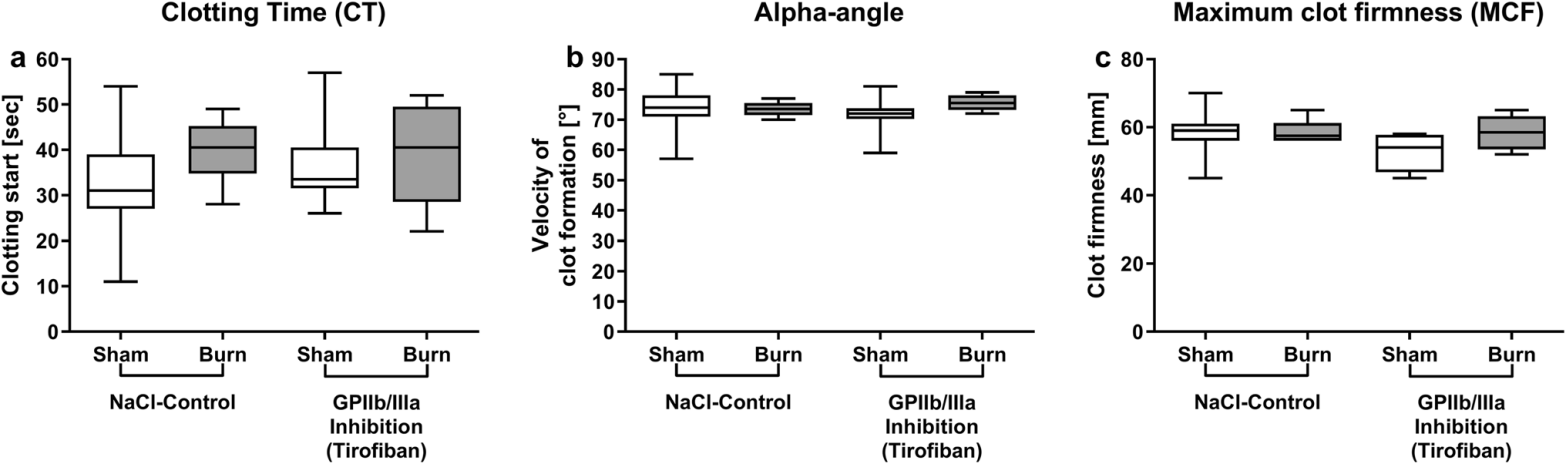
Early posttraumatic hemostasis is not changed after GPIIb/IIIa inhibition via tirofiban. The in vivo inhibition of GPIIb/IIIa via tirofiban prior to burn injury does not change parameters assessed by thromboelastometry. Clotting time, indicating the onset of clot formation, alpha-angle, indicating the velocity of clot formation, and maximum clot firmness (MCF), indicating the clot stability, were measured (**a**–**c**). Data are expressed as box plots of the median fluorescent intensity (MFI) ± Q1 and Q3, and whiskers indicate max and min data points. *n* = 6–8. *p < 0.05

GPIIb/IIIa inhibition did not significantly influence blood coagulation. CT, alpha-angle, and MCF in the tirofiban group did not differ significantly from the control group (Fig. 4a–c). The disruption of GPIIb/IIIa via tirofiban does not seem to influence ROTEM®-specific clotting parameters of platelets. The results are in line with findings of other authors, detecting no impact on ROTEM® parameters following GPIIb/IIa antagonist treatment [35].

### Disruption of fibrinogen annihilates effective hemostasis but interestingly the fibrinogenic pathway seems to be less affected than thrombocytes following burn trauma.

**Fig. 5:**
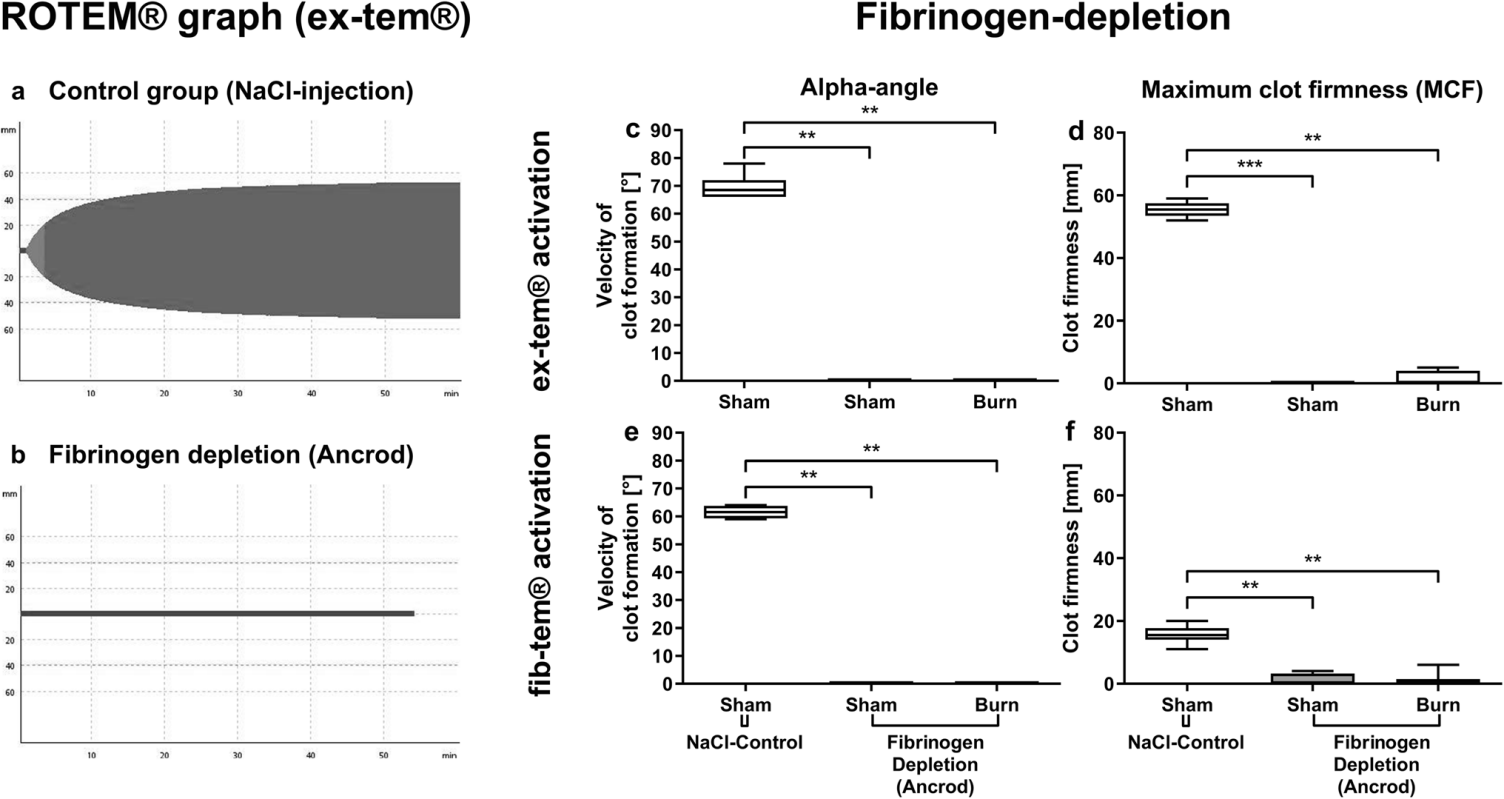
Disruption of fibrinogen annihilates effective hemostasis. The disruption of fibrinogen annihilates hemostasis but the fibrinogenic pathway seems to be less affected than platelets, as in the thromboelastometry analysis, the fib-tem® activation, which inhibits platelet contribution to the clot firmness, is slightly sustained compared to ex-tem® activation. Activation of coagulation was conducted with ex-tem® and fib-tem®. The graphs representing the clotting process in the control group (**a**) and in the fibrinogen depletion group (**b**) were displayed. The alpha-angle and maximum clot firmness were compared between all groups and interventions (**c**–**f**). Data are expressed as box plots of the median fluorescent intensity (MFI) ± Q1 and Q3, and whiskers indicate max and min data points. *n* = 6–8. *p < 0.05, **p < 0.01, ***p < 0.001

Following ancrod injection 1 h post trauma, rotational thromboelastometry (ROTEM®) via ex-tem® and fib-tem® analysis was conducted. Both reagents activate coagulation by tissue factor. Fib-tem® selectively excludes platelet contribution to clot formation via cytochalasin D. We analyzed the influence of ancrod on fibrinogen-dependent blood coagulation. A significant impact of ancrod on blood coagulation in both ex-tem®- and fib-tem®-induced blood coagulation was observed (Fig. 5c–f). Ancrod injection led to a significantly lower MCF and alpha-angle in the sham and burn groups. Consequently, the data indicates that fibrinogen inhibition via ancrod annihilates clot formation independent of trauma. Since fib-tem® excludes contribution of platelets to clot formation by reacting with cytochalasin D, it is optimal for analysis of fibrinogenic disruptions. By analyzing the difference between ex-tem® and fib-tem® MCF results, it is possible to identify the proper contribution of platelets to clot formation. The results (Fig. 5d, f) revealed the elimination of platelet-dependent and soluble coagulation factor-dependent clotting.

### Early posttraumatic hemostasis is not differed following PAR4 disruption

**Fig. 6:**
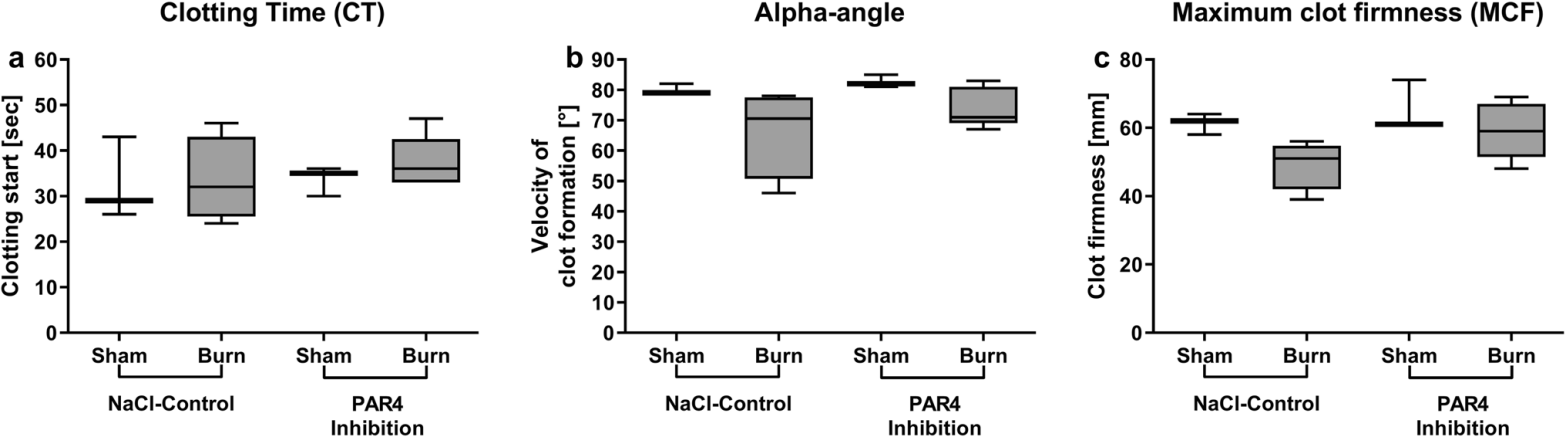
Early posttraumatic hemostasis is not differed following PAR4 disruption. The hemostasis assessed by thromboelastometry early post burn injury is not affected by PAR4 disruption. The clotting time (CT) (**a**), alpha-angle (**b**), and the maximum clot firmness (MCF) (**c**) were depicted. Data are expressed as box plots of the median fluorescent intensity (MFI) ± Q1 and Q3, and whiskers indicate max and min data points. *n* = 6–8. *p < 0.05

Blood coagulation 1 h following trauma did not show a significant impact of PAR4 disruption on hemostatic parameters in pre- and posttraumatic conditions compared to non-injected animals. CT, alpha-angle, and MCF did not differ significantly following PAR4 inhibition compared to the control group (Fig. 6a–c). These observations correspond to data gained from Mende et al., where disruption via tcY-NH_2_ did not change blood coagulation significantly in ROTEM® measurements [32]. As significant impact of PAR4 disruption in blood coagulation has been described in knockout mice [32], we believe that these different results may be due to the impact and dosage of the applied agent.

## Discussion

The immunological role of platelets is not yet fully understood. However, increasing numbers of studies concentrate on potential therapeutic use of pharmacologic targets on platelets to regulate the immune response [36]. In previous work, we revealed that platelets augment the activation of CD4^+^ Tregs in an early phase after trauma [14]. The mechanisms, however, remained unclear. Recently, it was shown that TNFR2- and TLR4-dependent pathways are modulating the activation of CD4^+^ Tregs following trauma [37]. In this work, we tested the hypothesis that platelets modulate CD4^+^ Tregs posttraumatically in a GPIIb/IIIa-, fibrinogen-, or PAR4-dependent manner*.*

### CD4^+^ Treg activation

Both ZAP-70 and PKC-Θ are downstream mediators of the T cell receptor (TCR) [34, 38]. ZAP-70 KO mice clinically reveal impaired CD4^+^ Treg function [39]. PKC-Θ in T effector cells propagate T cell function [34], whereas in CD4^+^ Tregs, their increased expression serves as a negative feedback loop [40]. The clinical effect of PKC absence in CD4^+^ Tregs is not fully elucidated [33, 40, 41] but given its upregulation, including the phosphorylated form, it makes it well suitable to measure Treg activation [8].

### GPIIb/IIIa hinders constant activation of local CD4^+^ Tregs via platelets

In our experiments, we used two different approaches to block the GPIIb/IIIa receptor. Firstly, we conducted a direct intervention to block the receptor via tirofiban. Secondly, we disrupted the most important ligand of the receptor: fibrinogen. GPIIb/IIIa plays an important role in hemostasis. The receptor also binds to different bacterial proteins, indicating that it might have a role in an immunologic activation of platelets [42]. Moreover, it is essential for the interaction between platelets and neutrophils [6]. We detected that neither GPIIb/IIIa nor fibrinogen inhibition in large influenced the systemic activation of CD4^+^ Tregs, as can be seen in the unaltered activation in the spleen. However, the basic activity level of CD4^+^ Tregs locally in the draining lymph nodes is increased following GPIIb/IIIa and fibrinogen inhibition.

Altogether, the local immune response shows that the basic activity of CD4^+^ Tregs is diminished by GPIIb/IIIa receptor engagement. The extent to which CD4^+^ Tregs become activated early after burn trauma is unchanged. Paradoxically, the direct blockage has a lower effect than the blockage of the ligand, fibrinogen.

GPIIb/IIIa is the most abundant receptor and plays a major role in platelet activation [15, 18]. Therefore, the GPIIb/IIIa receptor has mainly prothrombotic and pro-inflammatory actions, although several anti-inflammatory mechanisms are assumed [42]. The inhibition of GPIIb/IIIa led to an increased basic activation of CD4^+^ Tregs within the draining lymph nodes. We believe that there are two reasons for this. Platelets are cells with a limited ability to react on stimulation due to the lack of a nucleus. We suggest that after GPIIb/IIIa receptor engagement and subsequent thrombotic and inflammatory activation of the platelets, they then exhaust as cells without a nucleus due to their limited capabilities to generate new proteins. Platelets interact with CD4^+^ Tregs and augment their posttraumatic activation [14]. This augmentation takes place in the local draining lymph nodes and dampens the immunologic response in the early phase after burn trauma but is hindered when GPIIb/IIIa is activated. When GPIIb/IIIa is activated, pro-inflammatory mediators such as CXCL4, for example, are released [43] and CD40L expression stimulating endothelial cells are upregulated in a pro-inflammatory manner [20, 43]. Moreover, studies revealed protective effects of GPIIb/IIIa inhibition in different inflammatory models [44]. Our results support these findings and provide a potential mechanistic explanation. The anti-inflammatory effect of GPIIb/IIIa inhibition might be due to higher CD4^+^ Treg activity. The threshold for a systemic effect seems to be too low, as we observed no effect in splenic CD4^+^ Tregs revealed in PKC-θ expression. It has to be mentioned that the increase of non-phosphorylated ZAP-70 in the spleen could indicate that our model is underpowered in this regard. However, the phosphorylated form of ZAP-70 was unchanged. Therefore, we firmly assume the threshold for a systemic effect is too low.

Fibrinogen plays an important role in immunothrombosis, as it functions as a linking protein between platelets themselves as well as between platelets and neutrophils. An insufficient amount of fibrinogen leads to an inadequate response to pathogen infection [45, 46]. We suggest that following fibrinogen impairment and following GPIIb/IIIa inhibition, less platelets get activated and trapped into thrombi at the site of injury and therefore circulate more freely into the blood stream. We assume that the interaction between platelets and CD4^+^ Tregs is more frequent if free circulation is ensured. If fibrinogen is depleted, interaction with circulating cells in the blood stream outside the thrombus is possible. This is reflected by the increased basic activity of CD4^+^ Tregs in the draining lymph nodes and to a much lesser extent seemingly also in the spleen. Further studies need to be conducted to determine where exactly the interaction takes place.

### Platelets diminish the posttraumatic CD4^+^ Treg activation in a PAR4-dependent manner

PAR4 is expressed among others on platelets, leukocytes (monocytes, neutrophils, and T cells [47, 48]), smooth muscle, and endothelial cells [25, 26]. PAR4 serves as a crucial activator in inflammation besides hemostatic effects [49]. PAR4 inhibition leads to decreased recruitment of T cells [32]. PAR4 is an important player in immunothrombotic inflammatory interactions [32, 49] and if expressed positively on platelets, it regulates thrombin-dependent recruitment of pro-inflammatory leukocytes to platelet surface on thrombi via P-selectin [50]. Our results indicate that PAR4 activation prevents the posttraumatic activation of CD4^+^ Tregs in the local draining lymph nodes. Our results support the view of platelet PAR4 activation being a pro-inflammatory trigger that leads to increased T effector cell recruitment behavior [32] and as revealed negatively regulates the immunosuppressive CD4^+^ Treg activation following trauma. Among others, PAR4 is known to be expressed on T cells [49]. Recent findings indicate a higher CD4^+^ Treg activity following PAR4 inhibition [28]. To determine whether the effect seen in our data is platelet dependent, we compared the effect of inhibition of PAR4 on CD4^+^ Treg activation in presence and absence of platelet-rich plasma (PRP) in vitro. Our results indicate a platelet-dependent mechanism as the activation of CD4^+^ Tregs is higher when PAR4 is blocked in the presence of PRP. A limitation of this study is potential bystander effects under PAR4 inhibition by, e.g., endothelial cells. The results indicate that PAR4 expressed on platelets represent an important pro-inflammatory switch in the early posttraumatic phase.

Further, it suggests diverse activation of receptors on platelets. We believe platelets can act in an immunologic and a hemostatic manner, depending on selective receptor activation. Several authors showed that the activation of platelet TLR receptors did not lead to platelet aggregation, although it is known that TLR activation plays an important role, e.g., in atherosclerosis [42]. Several mechanisms of how platelets may be able to react differently to altering immunologic activations are suggested [6, 51]. It is hypothesized that platelets—according to the manner of stimulation—either secrete granules with different compositions containing cytokines or release only a certain fraction of proteins in a kiss-and-run technique when they merge with the cell membrane only for a very short time. If the manner of granule release is not altered, it is even suggested that platelets create certain proteins de novo after certain receptor stimulation [6, 51]. Although parts of these hypotheses are not yet fully proven, we are of the opinion that our data fits into this picture. Since a greater influence of PAR4 inhibition in draining lymph nodes was observed than in spleen cells, we postulate that posttraumatic interactions between CD4^+^ Tregs and platelets via PAR4 might be of more importance in local inflammation, than in the systemic immune response.

### Disruption of fibrinogen annihilates effective hemostasis

The disruption of fibrinogen annihilates hemostasis. Ex-tem® and fib-tem® both initiate coagulation via tissue factor; however, fib-tem® inhibits platelet contribution to the clot firmness, so that only the contribution of fibrinogen to the clot is displayed. Therefore, if the MCF of ex-tem® is subtracted from fib-tem®, it is possible to identify the contribution of platelets to the MCF. The results reveal that both platelet-dependent and soluble coagulation factor-dependent clotting are almost entirely disabled, which proves that platelets can circulate freely as they are not trapped in thrombi. As discussed above, we believe this enables more frequent contact to CD4^+^ Tregs in the blood stream.

### Early posttraumatic hemostasis is not affected by GPIIb/IIIa or PAR4 impairment

The disruption of PAR4 activation impaired immunologic functions whereas hemostatic functions of platelets were unchanged. The evaluation of tirofiban via ROTEM® analysis is limited, since impaired hemostasis via tirofiban cannot be detected by ROTEM® analysis [35] and in other studies using the same dose of tcY-NH_2_ did not significantly change ROTEM® parameters [32]. Platelets have hemostatic and immunologic functions. We hypothesize that the doses of both tirofiban and tcY-NH_2_ might be too low to have an impact on hemostatic functionality, as it is unlikely that they have no effect, given their well-reported hemostatic functions. However, it seems that the doses applied here disrupt the proper immunologic functionality of platelets following trauma.

## Conclusion

Our results indicate that the interplay between CD4^+^ Tregs and platelets is modulated via GPIIb/IIIa-, fibrinogen-, and PAR4-dependent pathways: the baseline activity of CD4^+^ Tregs is modulated by GPIIb/IIIa-, fibrinogen-, and PAR4-dependent platelet interaction. In addition, our observations suggest a crucial role for PAR4-dependent interactions of platelets and CD4^+^ Tregs following trauma. PAR4-dependent pathways modulate the posttraumatic activity of CD4^+^ Tregs. We suggest that the free circulation of platelets, as opposed to being bound in thrombi, ensures stimulation of CD4^+^ Tregs. Our findings demonstrate the importance of differential platelet functions, which contribute to the interaction of platelets and CD4^+^ Tregs. We suggest that platelets can act either in a hemostatic or in an immunologic way after trauma. Our findings provide a better understanding of the complex posttraumatic interplay of CD4^+^ Tregs and platelets, being a prerequisite in the development of reliable immunomonitoring tools and immunomodulatory drugs following trauma-induced injury.
